# The “Texas State Board of Health”

**Published:** 1899-09

**Authors:** 


THE "TEXAS STATE BOARD OF HEALTH."—A gentleman who had had the misfortune to lose his nose, was invited to dine with a family on a certain occasion. Anticipating criticism by sissy, the mother had said: "Now, sissy, when Mr. B.— comes, don't you say anything about his nose." At dinner mommer was horrified by sissy who exclaimed: "Mommer, you told me not to say anything about his nose; why, he ain't got any." The *New York Medical Record* says: "Many negroes in Bastrop, Brazos, Robertson and other counties in Texas are down with smallpox—the result of bad sanitary conditions following the recent floods in the Brazos valley;" and that "the State Board of Health has taken charge of the situation." How many more times will the JOURNAL have to publish the humiliating fact that Texas has no State Board of Health, never had, and it looks as if she never will have. Don't say anything more about "State Board of Health in Texas." We haven't any! The *Record* is advised that the spread of smallpox is not the result of unsanitary conditions, and that the outbreak is not due to such conditions. Both are the result of neglect of vaccination.

